# Event Source Modulates the Valence Focus of Optimism Bias: Evidence from Behavior and ERPs

**DOI:** 10.3390/brainsci16050479

**Published:** 2026-04-29

**Authors:** Chunsheng Wang, Baojing Zhang, Jun Ren

**Affiliations:** 1 School of Psychology, Zhejiang Normal University, Jinhua 321004, China; chunsheng.wang@zjnu.edu.com; 2 Key Laboratory of Intelligent Education Technology and Application of Zhejiang Province, Zhejiang Normal University, Jinhua 321004, China; jingbaozhang@163.com; 3 College of Education, Zhejiang Normal University, Jinhua 321004, China

**Keywords:** autonomous choice, optimism bias, late positive potential, self-relevance

## Abstract

**Highlights:**

**What are the main findings?**

- Under autonomous choice, optimism bias focuses on positive events. Under external imposition, it shifts to negative events.
- The LPP mirrors this behavioral pattern under autonomous choice. However, under external imposition, the LPP shows a valence-general self-bias. This reveals a dissociation between behavioral and neural responses.
- This dissociation suggests that different motivational processes may operate depending on the event source.

**What are the implications of the main findings?**

- This study provides preliminary electrophysiological evidence that the valence focus of optimism bias is not fixed. Instead, it may be an adaptive cognitive bias shaped by agency.
- These findings may inform future research on how perceived event sources could help modulate optimistic tendencies in real-world or clinical contexts.

**Abstract:**

**Background**: Optimism bias is the tendency to expect more positive and fewer negative events for themselves versus for others. However, it remains unclear whether the valence focus of this bias depends on how events unfold (i.e., through autonomous choice vs. external imposition). This study used behavioral and ERP measures to investigate how the event source modulates the valence focus of optimism bias. **Methods**: Thirty participants completed a 2 (event source: autonomous choice vs. external imposition) × 2 (valence: positive events vs. negative events) × 2 (target: self vs. other) task while an EEG was recorded. The late positive potential (LPP) was analyzed as a neural index of self-relevance. **Results**: Behaviorally, autonomous choice preserved optimism bias for positive events (self > other, *p* = 0.023), whereas external imposition induced it for negative events (self < other, *p* = 0.006). Under autonomous choice, the LPP mirrored this pattern (enhanced for self-positive events, *p* = 0.038). Under external imposition, the LPP showed only a valence-general self-bias (main effect of target, *p* = 0.014). **Conclusions**: These preliminary findings suggest that event sources may influence the valence focus of optimism bias. Under autonomous choice, the bias is positive-focused. Under external imposition, it shifts to negative-focused. This dissociation between behavioral and neural patterns is consistent with dual patterns in optimism bias expression, though further research is needed.

## 1. Introduction

Optimism bias is a common tendency. People see positive future events as more likely for themselves than for others. They see negative ones as less likely [1]. Researchers typically measure this bias by asking participants to estimate the likelihood of predetermined events happening to themselves versus similar others. This bias is influenced by factors such as major public events and controllability.

Optimism bias appears in many domains, such as health, academics, and finance [2]. Neuroimaging studies have revealed that this bias links to stronger brain activation in the rostral anterior cingulate cortex and amygdala when people process positive self-relevant information [3]. ERP studies also show that self-referential processing increases LPP amplitudes [4,5]. Researchers have observed a “self-positivity” bias: the LPP is larger for self-positive than for self-negative information. This pattern is not always found for other-referential information [6]. Together, these findings suggest that the brain prioritizes positive information linked to the self.

However, these existing paradigms use events with unclear causes. In reality, future events can come from autonomous choice (e.g., starting an investment) or external imposition (e.g., a pandemic). This ambiguity may lead people to guess the event source, which can affect their estimates. Evidence suggests that the event source matters. For example, externally imposed events like the COVID-19 pandemic reduce optimism bias [7]. This gap is important because real-world events rarely happen in a causal vacuum. People constantly face both self-chosen and imposed outcomes. Understanding how event sources shape optimism bias is essential for both theories of judgment bias and interventions that promote realistic expectations. Therefore, it is necessary to distinguish event sources to see how they affect the valence of optimism bias.

To address this gap, we draw on two complementary theories: cognitive dissonance theory and self-determination theory. Cognitive dissonance theory suggests that people adjust their attitudes to match their choices. This enhances the value of chosen outcomes [8,9]. Self-determination theory adds that autonomous choice satisfies the basic need for autonomy. This fosters intrinsic motivation and responsibility [10]. Integrating these ideas, we reasoned that autonomous choice could strengthen the psychological link between the self and events. Theoretically, this might happen through two routes: increasing self-relevance and psychological ownership or satisfying the need for autonomy [8,10]. Based on this reasoning, we formulated the following predictions: Under autonomous choice, for positive events, a stronger self-event link should make people more willing to embrace positive outcomes. Hence, the optimism bias (self > other) for positive events will be enhanced compared to a baseline or external condition. For negative events, a stronger self-event link may increase perceived responsibility, leading people to face negative outcomes more realistically. Therefore, the optimism bias for negative events (self < other) will be reduced or absent. In contrast, externally imposed events lack this self-referential foundation. Weaker self-linkage might reduce the bias toward externally imposed positive events. However, for imposed negative events, it may trigger psychological reactance, thereby lowering perceived personal risk.

We tested these hypotheses using event-related potentials (ERPs), focusing on the late positive potential (LPP). The LPP is a sustained positive ERP wave. It starts about 300–400 ms after stimulus. It is largest at parietal sites. It is reliably enhanced by motivationally salient and self-relevant stimuli [5,11,12]. Critically, prior research shows that the LPP is especially sensitive to self-positive information. Self-relevant positive stimuli evoke larger LPPs than self-negative or other related stimuli [5,6,13]. Thus, the LPP is a suitable neural marker for examining how the event source affects the valence-specific processing of future events. Based on these findings, we predicted that under autonomous choice, self-positive outcomes would elicit larger LPPs than other positive outcomes [5,6,13].

To test these hypotheses, we recorded an EEG while participants rated the likelihood of future events. We used a 2 × 2 × 2 within-participant design. The factors were event source (autonomous choice vs. external imposition), valence (positive vs. negative), and target (self vs. other). We focused on the LPP as our primary neural measure.

**Hypothesis 1 (replication of classic optimism bias).** 
*Regardless of the event source, we expected a significant Valence × Target interaction. Specifically, for positive events, participants would rate them as more likely for themselves than for others (self > other). For negative events, the opposite pattern would emerge (self < other) [1].*

**Hypothesis 2 (autonomous choice condition).** 
*Behaviorally, we predicted that under autonomous choice, the optimism bias for positive events would be present (self > other, possibly even stronger), whereas for negative events, it would be reduced or absent (no significant self-other difference). At the neural level based on prior LPP findings [5,6,13], we predicted that self-positive events would elicit larger LPPs than other positive events under autonomous choice.*

**Hypothesis 3 (external imposition condition).** 
*Behaviorally, we focused on negative events. We predicted that under external imposition, the optimism bias for negative events would be enhanced (self < other, with a larger difference) compared to the classic pattern, while positive events would show no significant self–other difference. At the neural level, we explored whether a valence-general self-bias (main effect of target) would emerge, as suggested by automatic self-referential processing.*

By systematically manipulating the event source, this study aims to extend our understanding of optimism bias—from a static, trait-like phenomenon to a dynamic, context-dependent cognitive bias. The findings may have implications for understanding how individuals maintain positive self-regard in the face of both self-chosen and externally imposed life outcomes.

## 2. Materials and Methods

### 2.1. Participants

Thirty Chinese university students (22 female, 8 male) took part in this study. They were aged 19–22 years (*M* = 20.13, *SE* = 0.14). The sample size was estimated using G*Power 3.1.9.7 [14]. With parameters ($\eta_{p}^{2}$ > 0.1, *β* = 0.95), the required sample was 23 participants. All participants had reported normal or corrected-to-normal vision. None had a history of neurological or psychiatric disorders. They all gave written informed consent before the experiment.

### 2.2. Stimuli, Design, and Procedure

Stimuli: The stimulus set consisted of 108 future event phrases (54 positive, 54 negative). The phrases were adapted from Weinstein [1]. To ensure relevance for Chinese university students, we generated additional items. These covered domains such as academic achievement, interpersonal relationships, health, and career development. All phrases were concrete and imaginable (the complete set of 108 event phrases (54 positive, 54 negative) is listed in Appendix A). Each phrase was presented twice: once for the self and once for others. This resulted in 216 trials per participant.

Design: This task builds on established paradigms. In these paradigms, random outcomes (e.g., die rolls, lottery draws) are used to assign a target (self/other) and manipulate an event source [15,16,17]. The study employed a 2 × 2 × 2 within-subject design. The factors were event source (autonomous choice vs. external imposition), valence (positive events vs. negative events), and target (self vs. other). All procedural steps were identical across the eight experimental conditions. Thus, cognitive load was balanced and did not systematically confound the comparisons.

The event source was manipulated by who made the choice. In the autonomous choice condition, participants actively selected an event from two masked options (one positive, the other negative). In the external imposition condition, they waited while a simulated “other” made the selection for them. We used masked options to prevent participants from selecting positive events based on valence. Otherwise, the autonomy manipulation would be confounded with a pre-existing valence preference. Participants were told that one option was positive and the other negative. However, they did not know which was which until after the choice. Immediate feedback after each selection showed the valence of the chosen option. This allowed participants to infer the valence of the unchosen option. Thus, the autonomy manipulation reflected the act of choosing itself, not a preference for positive outcomes. We manipulated choice ownership, not event controllability. Even trivial choices induce ownership [18]. Events were randomly assigned, so any controllability variability was balanced. Valence was manipulated by the type of event phrase. The target was manipulated by a die roll outcome: for half of the participants, high numbers (4–6) resulted in the self and low numbers (1–3) in others; the other half followed the reverse rule. Each phrase was presented twice—once for the self and once for others—evenly split between the autonomous choice and external imposition conditions and counterbalanced across participants, resulting in 27 trials per condition and 216 trials per participant. The dependent variable was the likelihood rating of each event, measured on a visual analog scale ranging from 0% (not at all likely) to 100% (extremely likely).

Procedure: Upon arrival, participants signed the informed consent form. Then they were led into a soundproof lab. The experimenter explained the procedure and rules in detail. All instructions were also presented on the screen. To ensure task comprehension and reduce cognitive load, participants completed a practice session of 8 trials covering all conditions before the formal experiment.

**Figure 1:**
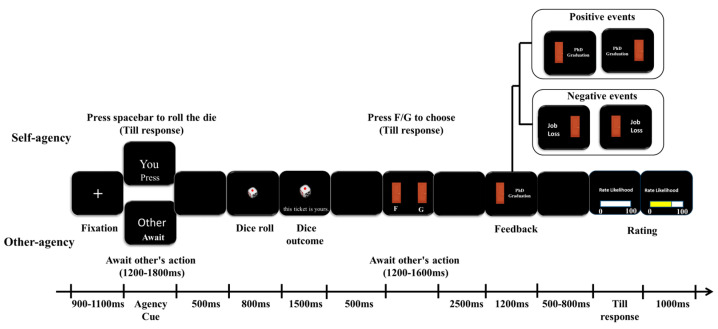
Experimental procedure: Participants first saw a role cue indicating whether they would be the active chooser (autonomous choice condition) or passive receiver (external imposition condition). A die roll then determined the target (self/other; mapping counterbalanced). In the autonomous choice condition, participants actively pressed the spacebar to roll the die; in the external imposition condition, they waited for a simulated “other” to press the key. Next, participants were presented with two masked options (one positive, one negative), unaware of their identities until after the choice. In the autonomous choice condition, they actively selected one by pressing F or G; in the external imposition condition, the computer randomly selected one on their behalf. After selection, the chosen event phrase was displayed, and participants rated the likelihood of it occurring to the designated target on a 0–100% visual analog scale. To accommodate ERP data collection, blank screens were inserted after key events, and fixation durations were jittered (900–1100 ms).

Each trial consisted of three sequential phases (see Figure 1), corresponding to the stages outlined in the main text.

1. Role Assignment: A trial began with a central fixation cross presented for 900–1100 ms. Then, a role cue appeared (self or other). Participants were told that “other” meant a typical university student of the same age and gender as themselves, not a specific known person. This cue signaled the forthcoming condition: autonomous or externally imposed.
2. Target Determination: A virtual die roll determined whether the upcoming event would apply to the self or to a similar other. The mapping was counterbalanced as described above. In the autonomous choice condition, participants actively pressed the spacebar to initiate the die roll. In the external imposition condition, they waited while a simulated “other” performed the key press after 1200–1800 ms. Upon the key press, a feedback screen (1000 ms) showed “Choose” turning yellow (autonomous choice) or “Await” turning red (external imposition). A dice animation was then shown for 800 ms, followed by the dice outcome displayed for 1500 ms.
3. Event Selection and Likelihood Estimation: After a 500 ms ISI, two masked options appeared. One option was a positive event, the other a negative event. The identities (which was positive, which was negative) were unknown until after the choice. In the autonomous choice condition, participants actively selected one of the two options by pressing the F or G key. In the external imposition condition, the simulated “other” selected one option on their behalf within 1200–1600 ms. After selection, the chosen event phrase was displayed for 1200 ms (feedback) after a 2500 ms ISI. Following a final ISI (500–800 ms), participants then estimated the likelihood that this event would occur to the designated target (self or other) by clicking on a horizontal visual analog scale ranging from 0% (not at all likely) to 100% (extremely likely). Upon clicking, a dynamic yellow fill extended from the scale’s origin to the selected position, providing visual feedback for 1000 ms.

Apparatus: Participants were seated approximately 80 cm from a 23-inch LCD monitor (Dell P2317H; Dell Inc., Round Rock, TX, USA; refresh rate: 60 Hz; resolution: 1920 × 1080). All stimuli were presented on a silver background with a central black region of 800 × 600 pixels.

### 2.3. Statistical Analysis and EEG Preprocessing

Statistical analyses were performed with SPSS 26.0 (IBM, Armonk, NY, USA). Behavioral data (likelihood ratings) and LPP amplitudes were analyzed using repeated-measures analyses of variance (ANOVAs) with factors of event source (autonomous choice vs. external imposition), valence (positive vs. negative), and target (self vs. other). Because all within-subject factors had only two levels, sphericity was automatically satisfied; therefore, no correction was applied. For the overall 2 × 2 × 2 ANOVA, the critical Valence × Target interaction was a single pre-specified test; no correction was applied. For the two follow-up 2 × 2 ANOVAs (autonomous choice and external imposition conditions), we applied Holm–Bonferroni correction to control for two comparisons. For the subsequent paired *t*-tests that decomposed the significant interactions, we report uncorrected *p*-values, as these tests are protected by the initial interaction test [19]. Both corrected and uncorrected *p*-values are reported in the Section 3 for transparency. Effect sizes are reported as partial eta-squared ($\eta_{p}^{2}$) for ANOVAs and Cohen’s *d* for *t*-tests.

Electroencephalographic (EEG) activity was recorded from 64 scalp sites using Ag/AgCl electrodes (EasyCap, Herrsching, Germany) placed in accordance with the international 10–20 system. The recording setup employed BrainAmp amplifiers (BrainProducts GmbH, Munich, Germany) controlled via BrainVision Recorder software (version 1.20). During acquisition, signals were online bandpass-filtered between 0.05 and 100 Hz and digitized at a sampling rate of 500 Hz. Electrode impedance was maintained below 5 kΩ. The ground electrode was positioned at AFz, while the left mastoid (TP9) served as the online reference. All signals were subsequently re-referenced offline to the averaged mastoids (TP9 and TP10).

Offline processing was conducted in MATLAB 2016b (MathWorks, Natick, MA, USA) using EEGLAB 14.1.2 [20]. Continuous data were bandpass-filtered with cutoff frequencies of 0.1 Hz (high-pass) and 30 Hz (low-pass). For the analysis of the late positive potential (LPP), data were segmented into epochs spanning from 200 ms pre- to 1000 ms post-stimulus onset, time-locked to the feedback stimulus. A baseline correction was applied using the 200 ms pre-stimulus interval. Artifact removal involved both manual inspection and automated correction: epochs exhibiting excessive drift or amplitude exceeding ±300 μV were discarded [13], while ocular artifacts were corrected via independent component analysis (ICA). After artifact rejection and removal of incorrect behavioral responses, an average of 204.9 trials (SD = 14.8) remained per participant (94.9% of the original 216 trials). The number of retained trials did not differ significantly across the eight experimental conditions (all *p* > 0.20). Finally, artifact-free epochs were averaged separately for each experimental condition.

Based on prior research showing that the LPP is reliably enhanced at parietal sites within a 400–800 ms time window by self-relevant and motivationally salient stimuli [5,11,12], we analyzed the mean LPP amplitude at electrode sites (P1, Pz, P2) in this time window. For each participant, average waveforms were computed separately for each condition (Event Source × Valence × Target).

## 3. Results

### 3.1. Behavioral Results

Overall analysis:

A 2 (event source: autonomous choice vs. external imposition) × 2 (valence: positive events vs. negative events) × 2 (target: self vs. other) repeated-measures ANOVA was conducted on likelihood ratings.

Main effects: There was a significant main effect of valence, *F*(1, 29) = 111.91, *p* < 0.001, $\eta_{p}^{2}$ = 0.79. Positive events (*M* = 67.63, *SE* = 1.83) were rated as more likely than negative events (*M* = 26.37, *SE* = 2.30). The main effect of event source was also significant, *F*(1, 29) = 37.89, *p* < 0.001, $\eta_{p}^{2}\;$ = 0.57. Participants rated events as more likely overall under autonomous choice (*M* = 48.62, *SE* = 0.70) than under external imposition (*M* = 45.37, *SE* = 0.84). The main effect of target was not significant, *F*(1, 29) = 0.09, *p* = 0.76, $\eta_{p}^{2}\;$ = 0.003.

**Figure 2:**
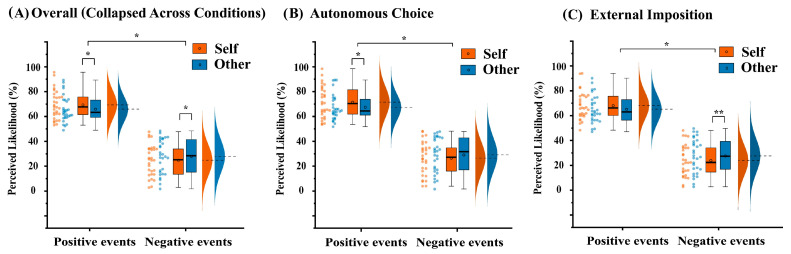
(**A**) Overall perceived likelihood for future positive and negative events for self vs. other. A significant Valence × Target interaction was observed. (**B**) Under the autonomous choice condition, the optimism bias was primarily driven by positive events. (**C**) Under the external imposition condition, a significant bias emerged for negative events. dashed lines/circles = mean; solid line = median. * *p* < 0.05, ** *p* < 0.01.

Valence × Target interaction: the significant Valence × Target interaction was significant, *F*(1, 29) = 5.79, *p* = 0.023, $\eta_{p}^{2}$ = 0.17, confirming the classic optimism bias [1]. Simple effect analyses showed that for positive events, participants rated them as more likely for themselves (*M* = 69.36, *SE* = 2.08) than for others (*M* = 65.89, *SE* = 1.92), *t*(29) = 2.17, *p* = 0.038, Cohen’s *d* = 0.40, 95% CI [0.20, 6.74]. For negative events, the opposite pattern emerged: participants rated them as less likely for themselves (*M* = 24.79, *SE* = 2.31) than for others (*M* = 27.94, *SE* = 2.49), *t*(29) = −2.35, *p* = 0.026, Cohen’s *d* = −0.43, 95% CI [−5.89, 0.41] (see Figure 2).

Other interactions: No other interactions reached statistical significance. Specifically, the Valence × Source interaction, *F*(1, 29) = 0.67, *p* = 0.42, $\eta_{p}^{2}$ = 0.02, the Target × Source interaction, *F*(1, 29) = 1.48, *p* = 0.23, $\eta_{p}^{2}$ = 0.05, and the three-way Valence × Source × Target interaction, *F*(1, 29) = 0.04, *p* = 0.85, $\eta_{p}^{2}$ = 0.001, were all not significant.

Follow-up analyses by event source:

To test whether the Valence × Target interaction was further modulated by the event source, we conducted separate 2 (valence) × 2 (target) repeated-measures ANOVAs for the autonomous choice and external imposition conditions (see Figure 2). To control for multiple comparisons across the two ANOVAs, we applied Holm–Bonferroni correction to the Valence × Target interaction tests.

Autonomous choice condition: The ANOVA revealed a significant main effect of valence, *F*(1, 29) = 106.86, *p* < 0.001, $\eta_{p}^{2}$ = 0.79. Positive events (*M* = 69.47, *SE* = 1.92) were rated as more likely than negative events (*M* = 27.78, *SE* = 2.33). The main effect of target was not significant, *F*(1, 29) = 0.78, *p* = 0.39, $\eta_{p}^{2}$ = 0.03. The Valence × Target interaction was significant before correction, *F*(1, 29) = 5.18, *p* = 0.030, $\eta_{p}^{2}$ = 0.15; after Holm–Bonferroni correction (for the two follow-up ANOVAs), the corrected *p* value remained 0.030, indicating a significant effect. Simple effect tests (uncorrected) were performed as planned comparisons within the autonomous choice condition. For positive events, participants rated them as more likely for themselves (*M* = 71.52, *SE* = 2.28) than for others (*M* = 67.42, *SE* = 1.90), *t*(29) = 2.40, *p* = 0.023, Cohen’s *d* = 0.44, 95% CI [0.60, 7.60]. For negative events, the self–other difference was not significant. Participants rated negative events similarly for themselves (*M* = 26.45, *SE* = 2.42) and for others (*M* = 29.10, *SE* = 2.53), *t*(29) = −1.58, *p* = 0.125, Cohen’s *d* = −0.29, 95% CI [−6.09, 0.78].

External imposition condition: The ANOVA revealed a significant main effect of valence, *F*(1, 29) = 113.29, *p* < 0.001, $\eta_{p}^{2}$ = 0.80. Positive events (*M* = 65.78, *SE* = 1.84) were rated as more likely than negative events (*M* = 24.95, *SE* = 2.32). The main effect of target was not significant, *F*(1, 29) = 0.50, *p* = 0.49, $\eta_{p}^{2}$ = 0.02. The Valence × Target interaction was also significant before correction, *F*(1, 29) = 5.73, *p* = 0.023, $\eta_{p}^{2}$ = 0.17; after Holm–Bonferroni correction, the corrected *p* value was 0.047, which remained significant. However, the pattern was reversed compared to the autonomous condition. Simple effect tests (uncorrected) revealed a significant bias for negative events: participants rated negative events as less likely for themselves (*M* = 23.13, *SE* = 2.29) than for others (*M* = 26.78, *SE* = 2.51), *t*(29) = −2.96, *p* = 0.006, Cohen’s *d* = −0.54, 95% CI [−0.58, 6.27]. For positive events, only a non-significant trend was observed, with self (*M* = 67.21, *SE* = 2.02) > other (*M* = 64.36, *SE* = 2.02), *t*(29) = 1.70, *p* = 0.099, Cohen’s *d* = 0.31], 95% CI [−6.16, −1.12].

### 3.2. LPP Results

To examine how the event source modulates the neural processing of self-relevant information, we conducted separate repeated-measures ANOVAs on LPP amplitudes. We did this for the autonomous choice condition and external imposition conditions. Each ANOVA included valence (positive vs. negative) and target (self vs. other) as factors. For the two follow-up ANOVAs, we applied Holm–Bonferroni correction to the Valence × Target interactions (as planned). For the external imposition condition, because we also had a specific prediction regarding the target main effect, we applied Bonferroni correction for the three within-ANOVA tests (α = 0.05/3 = 0.0167) to that effect. Simple effect tests are only reported when the interaction is significant [19].

**Figure 3:**
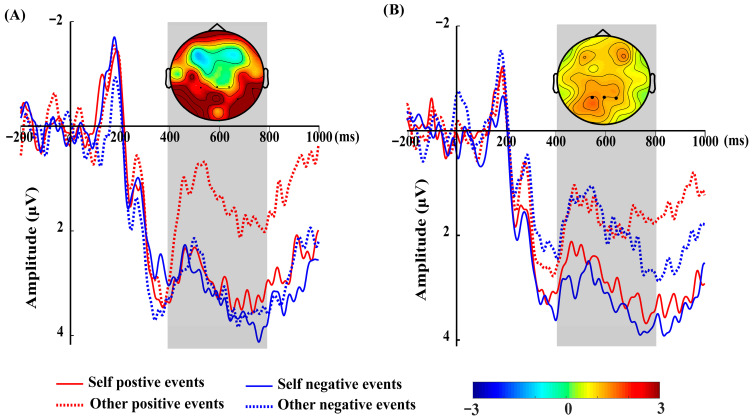
Grand-averaged ERPs over parietal electrodes (P1, Pz, P2) and difference topographies (400–800 ms). (**A**) Autonomous choice–valence main effect. Waveforms: positive > negative (collapsed across self/other). Topography: positive minus negative; warmer colors = larger LPP for positive events. (**B**) External imposition–target main effect. Waveforms: self > other (collapsed across valence). Topography: self minus other; warmer colors = larger LPP for self, indicating a valence-general self-bias.

Autonomous choice condition: A different profile emerged in the EEG data (see Figure 3 for grand-averaged waveforms). Under autonomous choice, the LPP showed a significant main effect of valence, *F*(1, 29) = 5.34, *p* = 0.028, $\eta_{p}^{2}$ = 0.16. Positive events (*M* = 2.55, *SE* = 0.70) elicited significantly smaller LPP amplitudes than negative events (*M* = 3.47, *SE* = 0.64). The main effect of target was not significant, *F*(1, 29) = 2.28, *p* = 0.142, $\eta_{p}^{2}\;$ = 0.07. Critically, the Valence × Target interaction was not significant after Holm–Bonferroni correction, *F*(1, 29) = 4.19, *p* = 0.0498 (uncorrected), the corrected *p* was 0.0996, $\eta_{p}^{2}$ = 0.13. Therefore, no further simple effect tests were performed for this condition.

External imposition condition: Under external imposition, the LPP showed a significant main effect of target, *F*(1, 29) = 6.87, *p* = 0.014, $\eta_{p}^{2}$ = 0.19. Self-related events (*M* = 3.15, *SE* = 0.52) elicited larger LPP amplitudes than other related events (*M* = 1.97, *SE* = 0.63). After Bonferroni correction (for three tests within this ANOVA), the corrected *p* was 0.042, which remained significant. No significant interaction was found, nor was the main effect of valence significant (both *p* > 0.10). This pattern indicates a valence-general self-bias under external imposition.

## 4. Discussion

This study integrated behavioral and EEG findings. Behaviorally, the event source changed the valence focus of optimism bias. Under autonomous choice, the bias focused on positive events (self > other). Under external imposition, it shifted to negative events (self < other). For LPP, the external imposition condition showed a valence-general self-bias (main effect of target). In the autonomous choice condition, the predicted LPP effect (self-positive vs. other positive) did not survive correction. These dissociable patterns suggest that the event source is an important factor in how optimism bias is expressed. Because we did not directly measure the underlying psychological processes, these findings should be interpreted as preliminary and hypothesis-generating [21].

Under autonomous choice, participants rated positive events as more likely for themselves than for others. For negative events, no self–other difference emerged. This pattern is consistent with theories of cognitive dissonance [8,9] and self-determination [10]. Choice behavior is oriented toward positive expectations. Thus, self-chosen positive outcomes receive privileged processing [22].

However, the LPP effect under autonomous choice was not significant after correction. Several factors may explain this. First, statistical power was limited. The effect size was moderate ($\eta_{p}^{2}$ = 0.13). With only 30 participants and correction for multiple comparisons, the study was underpowered to detect this effect. Second, ERP measures often have higher individual variability than behavioral ratings. Third, the average number of trials per condition was about 25, which is relatively low for ERP analysis and may have reduced the signal-to-noise ratio. Hence, we do not interpret the neural results in this condition. The behavioral effect stands alone. The null neural finding does not contradict the theory—it simply calls for replication with a larger sample.

Under external imposition, the optimism bias shifted to negative events (self < other). This pattern may reflect a self-protective strategy [7]. People may believe that negative events are less likely to happen to themselves. This belief could help preserve self-worth in uncontrollable contexts. Positive events lack this function, and their behavioral bias was absent. The LPP showed a significant main effect of target (self < other) regardless of valence. This indicates a valence-general self-bias. It suggests that even when behavior is strategically modulated, basic self-referential processing remains automatic and valence-independent. This dissociation aligns with research showing that self-relevance effects can persist at early neural stages despite contextual modulation [5,23,24]. Thus, the weakened self–event link under external imposition may leave a core, valence-independent self-representation [24,25].

Taken together, these findings reveal two distinct patterns. Under autonomous choice, a unified pattern emerged: behavior showed a positive-focused bias. Under external imposition, a dissociated pattern appeared: behavior showed a negative-focused bias, while neural responses retained a valence-general self-bias. This dissociation may reflect different cognitive systems—controlled versus automatic [26,27]. The behavioral bias under external imposition may involve strategic processes aimed at maintaining self-worth. the LPP self-bias may reflect automatic, obligatory self-referential processing that is less susceptible to contextual modulation [5].

Our findings extend the existing literature in several ways. First, event source is a new factor that shapes the valence focus of optimism bias. Second, the dissociation between behavior and neural responses provides preliminary evidence that agency may engage different levels of processing. Third, the valence-general LPP self-bias under external imposition supports the view that self-referential processing may be partially independent of valence evaluation [4,28,29]. All interpretations are speculative and need replication.

This study has several limitations. First, our sample consisted of Chinese university students. Generalization to other cultures and age groups require further investigation. Second, we used hypothetical events. Future studies should test real-world decisions. Third, the LPP is an index of self-relevance and motivational salience [5,11]. It does not directly measure psychological states. These include self-enhancement motives, self-referential processing strength, and defensive motivation. Moreover, the autonomous LPP effect was not significant, likely due to low statistical power. Future studies need larger samples. Future studies need larger samples. Fourth, we did not include a formal manipulation check (e.g., verifying participants’ perception of agency or understanding of masked choices). This is a limitation, and future research should incorporate such checks. Fifth, individual differences (e.g., trait optimism [30] and need for control [31]) were not examined. They could be included in future studies to identify who is most susceptible to these effects. Sixth, some extreme events (e.g., “terrorist attack”) may reduce the manipulation’s psychological reality. We note this limitation and suggest future studies compare extreme vs. everyday events or directly measure perceived controllability.

Future studies could explore developmental trajectories. Do children show similar modulation? Do older adults exhibit different patterns? Cross-cultural investigations would also be valuable. The meaning of autonomy versus external imposition may vary across cultures. Finally, neuroimaging studies with higher spatial resolution (e.g., fMRI) could identify brain regions supporting the observed dissociation here. In particular, researchers could examine the interplay between the default mode network and the frontoparietal control network. The default mode network is implicated in self-referential processing [32,33]. The frontoparietal control network is implicated in strategic, goal-directed processing [34]. In our study, all likelihood ratings were subjective estimates. To test whether agency still modulates optimism bias when participants receive objective, real-world probabilities, a future study could adopt the Sharot paradigm [2]. Framing the statistical feedback as resulting from the participant’s autonomous choice versus external imposition would directly test the event source manipulation, independent of subjective event controllability.

## 5. Conclusions

This study experimentally isolated the event source. The results show that agency modulates the expression of optimism bias. Under autonomous choice, the bias focuses on positive events. Under external imposition, it shifts toward negative events. These findings suggest that the valence focus of optimism bias is not fixed. Instead, it may be a cognitive bias shaped by how events occur. The dissociation between behavioral and neural patterns also highlights the value of multi-method approaches for examining the complex mechanisms underlying judgment biases.

## Data Availability

The data presented in this study are available upon request from the corresponding author due to privacy protection.

## Abbreviations

ERP	Event-related potentials
LPP	Late positive potential

## Appendix A

**Table A1:** Complete list of 108 future life event words (54 positive, 55 negative).

Positive Events		Negative Events	
1. Live in peace	28. Harmonious family	1. Be kidnapped	28. Family illness
2. Peaceful retirement	29. Financial prosperity	2. Be sued	29. Bereavement
3. Graduate on time	30. High salary	3. Be robbed	30. Interpersonal conflict
4. Retire on time	31. Exams passed	4. Be falsely accused	31. Become widowed
5. Financial freedom	32. Healthy family	5. Property stolen	32. Disease
6. Win lottery	33. Self-fulfillment	6. Fistfight	33. Romantic breakup
7. A good job	34. Good relationships	7. Sidelined at work	34. Lose job
8. Good grades	35. Good health	8. Get H1N1	35. Be laid off
9. Excellent grades	36. Salary increase	9. Loss	36. Conflict with teacher
10. Startup success	37. Ideal home	10. Legal dispute	37. Food poisoning
11. Meet expectations	38. Happy life	11. Marital separation	38. Career setback
12. Defense success	39. Realize life goals	12. Bone fracture	39. Attempt suicide
13. Travel abroad	40. Career success	13. Friend breakup	40. Conflict with classmates
14. Help from friends	41. Stable income	14. Get cancer	41. Lonely in old age
15. Gain recognition	42. Supervisor appreciation	15. Major illness	42. Suspend schooling
16. Achieve happiness	43. Respected by others	16. Marital failure	43. Delay graduation
17. Recognition	44. Smooth investment	17. Miscarriage	44. The wrong career
18. Gain good friends	45. Late-life joy	18. Family conflict	45. Accidental injury
19. No winter illness	46. Good studies	19. Traffic accident	46. Be cheated on
20. Good marriage	47. Own a house	20. Financial hardship	47. Earthquake
21. Job satisfaction	48. Healthy children	21. Fail an exam	48. House fire
22. Mentor help	49. Meet a good teacher	22. Terrorist attack	49. Die young
23. Happy marriage	50. Longevity	23. Appendicitis	50. Be scammed
24. Live past 80	51. Find a good job	24. Alzheimer’s	51. Jobless grad
25. Win honors	52. Find true love	25. Divorce	52. Have a stroke
26. Performance goals	53. Get promoted	26. Neighbor dispute	53. Midlife job loss
27. Family support	54. Intelligent children	27. Get lost	54. Go to prison
